# Motor Aspects of Handwriting Acquisition and Developmental Dysgraphia

**DOI:** 10.3390/children12070870

**Published:** 2025-06-30

**Authors:** Caroline Jolly

**Affiliations:** Laboratoire de Psychologie & NeuroCognition (LPNC CNRS 5105), Université Grenoble Alpes, Université Savoie Mont-Blanc, CNRS, LPNC, 38000 Grenoble, France; caroline.jolly@univ-grenoble-alpes.fr

Handwriting is a complex skill involving perceptual, motor, linguistic, and cognitive processes [1]. It requires several years of formal learning and training before complete acquisition. Its mastering is necessary for the subsequent acquisition of other skills, such as syntax, orthograph, or composition [1]. Graphomotor skills represent the prerequisite to handwriting [2]. Handwriting acquisition evolves from drawing of simple forms in young children to more and more complex tasks with age, until complete automation. Handwriting differs from drawing in that it is the translation of thoughts into linguistic representations. Once children write words, spelling and orthographic processes are also engaged [3]. Novice writers slowly write using single movements via visual control (retroactive control mode based on sensory feedbacks) and their speed profile displays constant acceleration and deceleration pulses, while skilled writers show only one movement pulse per stroke [4]. The control of fine handwriting movements in proficient writers is proactive, mainly based on internal motor programs. Handwriting automation occurs around the age of 9–10 and relies on the shift from the retroactive to the proactive control mode [1].

Despite correct learning and a sufficient level of practice, some children never master handwriting to a functional level [5]. Handwriting disorders are referred to as dysgraphia and concern 5–10% of school-aged children. If not treated, dysgraphia can have deleterious consequences on the further acquisition of other skills and on the child’s self-esteem. It is thus crucial to diagnose and handle these difficulties with appropriate rehabilitation protocols.

This Special Issue on “Motor learning of handwriting and developmental dysgraphia” presents nine contributions encompassing various aspects of handwriting acquisition and dysgraphia: three are reviews (Contributions 1 to 3) and six are research articles (Contributions 4 to 9). Part of the research articles concern developmental aspects of handwriting (Contributions 4 and 5), while the others bring new insights on the understanding of handwriting disorders (Contributions 6 to 9).

Bonneton-Botté and colleagues (Contribution 1) present a comprehensive review on handwriting intervention, both from an educational and clinical rehabilitation perspective. They focus more particularly on recent digital technologies to assist handwriting learning, to facilitate access to education, to increase children’s participation in learning activities, and/or to help diagnose and rehabilitate handwriting. A special emphasis is placed on technologies based on Artificial Intelligence (AI) as well as on devices that have already been introduced in teaching and rehabilitation programs. The existing methods for the diagnosis of dysgraphia, with a particular focus on digital methods, are reviewed in Danna et al. (Contribution 2). However, as emphasized in Contribution 1, the clinical tests that have been developed in various languages and/or alphabets to evaluate handwriting difficulties present a certain number of caveats. First, transferrability from one language to another is difficult, especially when the alphabets are different. Second, normative data for each school grade should be provided in each language. Third, many of these tests are partly subjective and depend on the evaluator’s expertise. Fourth, these tests focus on the written product but do not evaluate the gesture. Last but not least, none of these tests allows for the identification of the cause of handwriting deficits. For instance, as reviewed in Contribution 2, the handwriting deficits observed differ depending on the associated neurodevelopmental disorder. While the nature of the deficits is relatively clear in the case of dyslexia or developmental coordination disorder (DCD) (Contribution 2), a substantial uncertainty about handwriting skills in children with Attention Deficit Hyperactivity Disorder (ADHD) remains in the literature, as reviewed by Puyjarinet et al. (Contribution 3). This review highlights the need for high-quality future studies with more controlled inclusion criteria for the participants, namely comorbidities, ADHD subtype, or their medication status. This observation turns out to be valid for all neurodevelopmental disorders.

Given the wide heterogeneity of handwriting deficits, the recent development of digital technologies may facilitate handwriting acquisition, help in the diagnosis of dysgraphia and associated neurodevelopmental disorders, and provide relevant clues for personalized remediation protocols, as proposed in Contributions 1 and 2. A first interesting tool is a digitizing tablet and stylus with dedicated software, which allows for the quantification of many aspects of handwriting that cannot be measured clinically and which can be particularly relevant from an educational or rehabilitation perspective (Contributions 1 and 2). These tools and software will become more and more powerful thanks to the ongoing progress of AI. The perspectives about the development of a future universal standardized test for dysgraphia, combining computer and paper-and-pen tools are discussed in Contribution 2. More specifically, these authors propose a future tool that includes a combination of tasks targeting different skills, not only handwriting. Indeed, as highlighted by Maurer and Eckhart (Contribution 4), graphomotor skills encompass a set of psychomotor abilities that enable drawing and handwriting. The inclusion of graphomotor tasks in a diagnosis tool for dysgraphia thus makes sense, allowing for an earlier detection of “at-risk” children, and the comparison between different languages and alphabets. Other promising future tools are virtual reality (VR) and handwriting robots, as mentioned in Contribution 1. These technologies enhance the child’s engagement and can provide auditory or visual feedback during handwriting practice. However, the translation of these innovative and promising tools to education and clinic is still quite limited, in part due to the lack of acceptance of new technologies both from educational and rehabilitation professionals, as seen in Contribution 1.

Although having been explored for many years, several aspects of handwriting acquisition still remain poorly investigated. In Contribution 4, for instance, the authors explore the predictive contribution of gender, working memory, and motivation to handwrite on subsequent graphomotor skills and spelling, through a combination of tasks in children. They show that graphomotor skills predict spelling while considering gender and motivation. Girls exhibited higher performances in graphomotor skills, while boys tended to spell more accurately. This work underlines the importance of graphomotricity on non-motor aspects of handwriting. Similarly, the research article by Gerth and Festman (Contribution 5) focuses on another poorly investigated aspect of handwriting acquisition: the muscular control of handwriting movements. Indeed, handwriting requires the manipulation and fine control of a writing tool on a 2-dimensional surface. In adults, the distal joints (wrist and fingers) are mainly responsible for the fine movement control, while the proximal joints (shoulder and elbow) are essentially involved in maintaining the pen on the horizontal plane. The proximal muscles have been shown to function as stabilizers for the distal muscles during handwriting. In Contribution 5, the authors investigate the link between handwriting movements and upper limb muscle activity in children and adults using surface EMG (sEMG). They showed that the stabilizer function of the shoulder (trapezius muscle) in controlling handwriting movements is observed in adults but not in children, suggesting that it progressively develops during handwriting acquisition in children. Moreover, developing writers mainly use their proximal muscles to control the velocity of their handwriting movements, while skilled writers involve rather distal muscles to control their pen pressure on the surface.

The next three articles focus on handwriting difficulties, either from a diagnosis or a rehabilitation perspective. First, Loizzo et al. (Contribution 6) present an adaptation and validation of the gold-standard BHK test used for the diagnosis of dysgraphia in a sample of 283 Italian children from second to fifth grade. These authors found that 10% of the participants are above the cut-off for the quality score and can thus be considered as non-proficient writers, while 21.6% of the participants are considered as at-risk. A strong effect of gender is observed for the quality, but no effect of grade. Conversely, for the speed score, a strong effect of grade is observed but no effect of gender. The authors thus propose that Italian children with functional handwriting reach their final quality level by the end of the first grade, while children with dysfunctional handwriting may continue to improve their quality up to fifth grade. Interestingly, the authors again highlight the fact that the BHK must be part of a complete clinical evaluation and neuropsychological testing of the child, as mentioned above (Contributions 1 and 2).

The article by Lichtsteiner et al. (Contribution 7) investigates the effectiveness of PsychoMotor Therapy (PMT) in enhancing fine motor and handwriting skills in children with graphomotor impairment with and without Developmental Coordination Disorder (DCD). Indeed, PMT is a widely used treatment for children with graphomotor impairments in Switzerland, although there is a lack of scientific evidence to support its effectiveness in rehabilitating handwriting impairments. The authors found that PMT significantly improved fine motor skills in children with handwriting disorders (HDs) and DCD over a 5-month period, but no improvement was observed on handwriting fluency and consistency. This article thus highlights the importance of bringing additional therapeutic support to children with handwriting difficulties.

The first article by Vaivre-Douret and Lopez (Contribution 8) investigates the predictive validity of a previously validated pre-scriptural task for the diagnosis of handwriting disorders [6]. This task consists of tracing a line of cycloid loops, and was performed on a graphic tablet to analyze kinematic features. Gestural and postural parameters were captured with cameras and analyzed. The authors show that in children with handwriting disorders, gestural patterns were significantly less mature and associated with poorer quality, less fluid, and slower drawing. Moreover, good correlations between temporal and kinematic measures and the BHK scale were found. More specifically, number of strokes, total drawing time, in-air pause times, and number of velocity peaks showed very good sensitivity (88%) and specificity (74%) for diagnosing HDs. This cycloid loop task thus represents a robust and predictive tool to identify handwriting disorders in children before the acquisition of the alphabet. Following the recommendations given for the future development of a universal standardized digital test for the early diagnosis of handwriting difficulties (Contribution 2), such cycloid tasks appear particularly relevant and should be included.

Finally, the second article by Vaivre-Douret and Lopez (Contribution 9) addresses the question of the origin of the wide heterogeneity of handwriting disorders. To this end, the authors performed an in-depth transdisciplinary and complete approach using standardized clinical assessments to investigate neuropsychomotor, neuropsychological and oculomotor functions in children with handwriting disorders. Using this approach, they confirm the high heterogeneity among children with handwriting disorders and identified three subgroups: mild HDs, which are not detected by the BHK (26% of the children); moderate HDs (33% of the children); and dysgraphia, assessed by the BHK (41% of the children). Mild HDs are associated at a low frequency with disorders identified based on clinical evaluations, while dysgraphia appears to be associated with a high-frequency of co-occurring disorders, mainly oculomotor disorders (55% of the children). Moderate HDs are in between these two situations. Thus, the severity of the HDs may reflect distinct semiologies. This work again highlights the importance of performing a complete and transdisciplinary clinical evaluation in children presenting with HDs, in order to obtain a precise diagnosis and a personalized rehabilitation program. Moreover, the fact that some HDs are not detected using the BHK test, although the children are penalized by their poor handwriting at school, can be due to the short length and relative ease of the test. Indeed, the BHK test only lasts 5 min, and the children are not in a double-task situation when they pass it, in contrast to when in the classroom context. These results highlight the importance of proposing the use of several tasks for a longer duration (at least 20 min) for a more complete evaluation of handwriting and graphomotor skills, especially in the context of a future universal standardized test for dysgraphia as proposed in Contribution 2.

To conclude, this Special Issue highlights the wide heterogeneity of handwriting deficits in children and the importance of a multidisciplinary approach to more precisely evaluate the nature and the origin of these difficulties. Moreover, future fundamental research is also needed to further unravel certain less-explored aspects of handwriting acquisition.
